# Supplementary material for: Understanding and responding to COVID-19 in Wales: protocol for a privacy-protecting data platform for enhanced epidemiology and evaluation of interventions
Source: BMJ Open. 2020 Oct 21;10(10):e043010. doi: 10.1136/bmjopen-2020-043010 (PMC7580065; doi:10.1136/bmjopen-2020-043010)
Supplement: Author's manuscript [file bmjopen-2020-043010.draft_revisions.pdf]

BMJ Open is committed to open peer review. As part of this commitment we make the peer review history of every article we publish publicly available.

When an article is published we post the peer reviewers' comments and the authors' responses online. We also post the versions of the paper that were used during peer review. These are the versions that the peer review comments apply to.

The versions of the paper that follow are the versions that were submitted during the peer review process. They are not the versions of record or the final published versions. They should not be cited or distributed as the published version of this manuscript.

BMJ Open is an open access journal and the full, final, typeset and author-corrected version of record of the manuscript is available on our site with no access controls, subscription charges or pay-per-view fees (http://bmjopen.bmj.com).

If you have any questions on BMJ Open's open peer review process please email info.bmjopen@bmj.com

BMJ Open

Understanding and responding to COVID-19 in Wales: protocol for a privacy protecting data platform for enhanced epidemiology and evaluation of interventions.

Journal:	*BMJ Open*
Manuscript ID	bmjopen-2020-043010
Article Type:	Protocol
Date Submitted by the Author:	23-Jul-2020
Complete List of Authors:	Lyons, Jane; Swansea University Medical School, Population Data Science<br>Akbari, Ashley; Swansea University Medical School, Population Data Science<br>Torabi, Fatemeh; Swansea University Medical School, Population Data Science<br>Davies, Gareth; Swansea University Medical School, Population Data Science<br>North, Laura; Swansea University Medical School, Population Data Science<br>Griffiths, Rowena; Swansea University Medical School, Population Data Science<br>Bailey, Rowena; Swansea University Medical School, Population Data Science<br>Hollinghurst, Joseph; Swansea University Medical School, Population Data Science<br>Fry, Richard; Swansea University Medical School, Population Data Science<br>Turner, Samantha L.; Swansea University Medical School, Population Data Science<br>Thompson, Daniel; Swansea University Medical School, Population Data Science<br>Rafferty, James; Swansea University Medical School, Population Data Science<br>Mizen, Amy; Swansea University Medical School, Population Data Science<br>Orton, Chris; Swansea University Medical School, Population Data Science<br>Thompson, Simon; Swansea University Medical School, Population Data Science<br>Au-Yeung, Lee; Swansea University Medical School, Population Data Science<br>Cross, Lynsey; Swansea University Medical School, Population Data Science<br>Gravenor, Mike; Swansea University Medical School, School of Medicine<br>Brophy, Sinead; Swansea University Medical School, Population Data Science<br>Lucini, Biagio; Swansea University Medical School, Population Data Science

John, Ann; Swansea University Medical School, Population Data Science
Szakmany, Tamas; Cardiff University, Department of Anaesthesia, Intensive Care and Pain Medicine, Division of Population Medicine; Aneurin Bevan University Health Board, Critical Care Directorate
Davies, Jan and Chris
Davies, Chris
Thomas, Daniel; Public Health Wales
Williams, Christopher; Public Health Wales
Emmerson, Chris; Public Health Wales
Cottrell, Simon; Public Health Wales
Connor, Thomas; Cardiff University, School of Biosciences
Taylor, Chris; Cardiff University
Pugh, Richard; Glan Clwyd Hospital
Diggle, Peter; Lancaster University, Faculty of Health and Medicine; University of Liverpool, Epidemiology and Population Health
John, Gareth; NHS Wales Informatics Service,
Scourfield, Simon; NHS Wales Information Service
Hunt, Joe; NHS Wales Information Service
Cunningham, Anne Marie; NHS Wales Information Service
Helliwell, Kathryn; Welsh Government
Lyons, Ronan; Swansea University Medical School, Population Data Science

Keywords: COVID-19, EPIDEMIOLOGY, PUBLIC HEALTH

SCHOLARONE™
Manuscripts

I, the Submitting Author has the right to grant and does grant on behalf of all authors of the Work (as defined in the below author licence), an exclusive licence and/or a non-exclusive licence for contributions from authors who are: i) UK Crown employees; ii) where BMJ has agreed a CC-BY licence shall apply, and/or iii) in accordance with the terms applicable for US Federal Government officers or employees acting as part of their official duties; on a worldwide, perpetual, irrevocable, royalty-free basis to BMJ Publishing Group Ltd ("BMJ") its licensees and where the relevant Journal is co-owned by BMJ to the co-owners of the Journal, to publish the Work in this journal and any other BMJ products and to exploit all rights, as set out in our licence.

The Submitting Author accepts and understands that any supply made under these terms is made by BMJ to the Submitting Author unless you are acting as an employee on behalf of your employer or a postgraduate student of an affiliated institution which is paying any applicable article publishing charge ("APC") for Open Access articles. Where the Submitting Author wishes to make the Work available on an Open Access basis (and intends to pay the relevant APC), the terms of reuse of such Open Access shall be governed by a Creative Commons licence – details of these licences and which Creative Commons licence will apply to this Work are set out in our licence referred to above.

Other than as permitted in any relevant BMJ Author's Self Archiving Policies, I confirm this Work has not been accepted for publication elsewhere, is not being considered for publication elsewhere and does not duplicate material already published. I confirm all authors consent to publication of this Work and authorise the granting of this licence.

For peer review only

Understanding and responding to COVID-19 in Wales: protocol for a privacy protecting data platform for enhanced epidemiology and evaluation of interventions.

Jane Lyons, Data Science Building, Population Data Science, Swansea University Medical School, Singleton Park, Swansea, SA2 8PP, UK, J.Lyons@Swansea.ac.uk, 01792 513028 (corresponding author)

Ashley Akbari, Population Data Science, Swansea University Medical School, Swansea, UK

Fatemeh Torabi, Population Data Science, Swansea University Medical School, Swansea, UK

Gareth Davies, Population Data Science, Swansea University Medical School, Swansea, UK

Dr Laura North, Population Data Science, Swansea University Medical School, Swansea, UK

Rowena Griffiths, Population Data Science, Swansea University Medical School, Swansea, UK

Rowena Bailey, Population Data Science, Swansea University Medical School, Swansea, UK

Dr Joe Hollinghurst, Population Data Science, Swansea University Medical School, Swansea, UK

Dr Richard Fry, Population Data Science, Swansea University Medical School, Swansea, UK

Samantha Turner, Population Data Science, Swansea University Medical School, Swansea, UK

Dr Daniel Thompson, Population Data Science, Swansea University Medical School, Swansea, UK

Dr James Rafferty, Population Data Science, Swansea University Medical School, Swansea, UK

Dr Amy Mizen, Population Data Science, Swansea University Medical School, Swansea, UK

Chris Orton, Population Data Science, Swansea University Medical School, Swansea, UK

Simon Ellwood-Thompson, Population Data Science, Swansea University Medical School, Swansea, UK

Lee Au-Yeung, Population Data Science, Swansea University Medical School, Swansea, UK

Lynsey Cross, Population Data Science, Swansea University Medical School, Swansea, UK

Professor Mike Gravenor, Population Data Science, Swansea University Medical School, Swansea, UK

Professor Sinead Brophy, Population Data Science, Swansea University Medical School, Swansea, UK

For peer review only

Professor Biagio Lucini, Population Data Science, Swansea University Medical School, Swansea, UK

Professor Ann John, Population Data Science, Swansea University Medical School, Swansea, UK

Dr Tamas Szakmany, Cardiff University, Cardiff, UK

Jan Davies, Member of public, Swansea, UK

Chris Davies, Member of public, Swansea, UK

Professor Daniel Rh Thomas, Public Health Wales, Cardiff, UK

Dr Christopher Williams, Public Health Wales, Cardiff, UK

Chris Emmerson, Public Health Wales, Cardiff, UK

Dr Simon Cottrell, Public Health Wales, Cardiff, UK

Dr Thomas Connor, Cardiff University, Cardiff, UK

Professor Chris Taylor, Cardiff University, Cardiff, UK

Dr Richard Pugh, Betsi Cadwalader University Health Board, Rhyl, UK

Prof Peter J Diggle, Lancaster University, Lancaster, UK

Dr Gareth John, NHS Wales Informatics Service, Cardiff, UK

Simon Scourfield, NHS Wales Informatics Service, Cardiff, UK

Joe Hunt, NHS Wales Informatics Service, Cardiff, UK

Dr Anne Marie Cunningham, NHS Wales Informatics Service, Cardiff, UK

Kathryn Helliwell, Welsh Government, Cardiff, UK

Professor Ronan A Lyons, Population Data Science, Swansea University Medical School, Swansea, UK

Keywords: COVID-19, Data linkage, epidemiology, public health, evaluation

Word count: 2,196

For peer review only

ABSTRACT

Introduction

The emergence of the novel respiratory SARS-CoV-2 and subsequent COVID-19 pandemic has required rapid assimilation of population-level data to understand and control the spread of infection in the general and vulnerable populations, and to provide evidence to inform policy development and target interventions to at risk groups to prevent serious health outcomes. We aim to provide an accessible research platform to determine demographic, socioeconomic and clinical risk factors for infection, morbidity, and mortality of COVID-19, measure the impact of COVID-19 on healthcare utilisation and long-term health, and to enable the evaluation of natural experiments of policy interventions.

Methods and analysis

Two privacy-protecting population-level cohorts have been created and derived from multi-sourced demographic and healthcare data. The C20 cohort consists of 3.2 million people in Wales on the 1st January 2020 with follow up until 31st May 2020. The complete cohort dataset will be updated monthly with some individual datasets available daily. The C16 cohort consists of 3 million people in Wales on the 1st January 2016 with follow up to the 31st December 2019. C16 is designed as a counterfactual cohort to provide contextual comparative population on disease, health service utilisation, and mortality. Study outcomes will: a) characterise the epidemiology of COVID-19, b) assess socioeconomic and demographic influences on infection and outcomes, c) measure impact of COVID-19 on short term and longer-term population outcomes and d) undertake studies on the transmission and spatial spread of infection.

Ethics and dissemination

The Secure Anonymised Information Linkage (SAIL) independent Information Governance Review Panel (IGPR) has approved this study. The study findings will be presented to policy groups, public meetings, national and international conferences, and published in in peer-reviewed journals.

For peer review only

Strengths and limitations of this study

- Rapid access to multiple data sources on a complete population.
- Great variety of individual and household level data on demography, disease status, morbidity, mortality and viral genomics to support a wide range of studies on the evolution of the epidemic in Wales.
- Ability to support hierarchical analyses at varying geographical units: private residences, care homes, educational setting and healthcare facilities to examine spatial spread and transmission of SARS-CoV-2 to inform and evaluate targeting of interventions.
- However, routine data does not capture data on some important aspects, such as quality of life

For peer review only

INTRODUCTION

Understanding and controlling the COVID-19 pandemic is a rapidly changing, complex issue that requires near real-time local data, analyses, modelling and multidisciplinary team science to devise, implement and evaluate a wide variety of inter- and cross-sectoral interventions to minimise population harm.[1]

As the pandemic evolves a wide range of issues need to be considered including; the spread of infection in the general and vulnerable populations; health service resilience; indirect harm minimisation; and effectiveness of control policies and interventions.

Responding to this challenge, the Welsh Government created a COVID-19 Technical Advisory Group (TAG) to provide rapid assimilation of available evidence and guide analysis of data to inform policy development and appraisal. Insight from linked data is seen as being essential to understand the evolving epidemic. TAG commissioned the support of analyses conducted through the Secure Anonymised Information Linkage (SAIL) Databank (www.saildatabank.com) to formulate evidence and advice to underpin its work in responding to COVID-19.[2-5] SAIL is a state of the art, remotely accessible, privacy-protecting system, accredited under the Digital Economy Act, which holds and provides access to linked de-identified data from multiple sources at individual, household and multiple ecological levels, for the population of Wales. The SAIL Databank has previously supported numerous types of clinical and population studies, including cohorts, evaluations of natural experiments and embedded trials.[6-13]

For peer review only

This paper describes the development of two population-based cohorts in Wales, derived from multiple data sources to provide near real-time, in-pandemic intelligence and analytics to TAG in relation to the following broad objectives:

Primary objectives

a) Determine demographic, socioeconomic and clinical risk factors for infection, morbidity, and mortality related COVID-19;
b) Determine risk of COVID-19 infection and outcomes in occupational groups; and
c) Measure the population impact of COVID-19 on healthcare utilisation.

Secondary objectives

a) Create a platform to enable the evaluation of policies and interventions aimed at controlling the epidemic, whether clinical or non-pharmaceutical in nature; and

b) Provide access to these derived population-based cohorts and linked data sources to organisation and people with relevant skills and expertise within the NHS, academia and government.

METHODS

Study design and population

The cohorts were derived from de-identified linked data from the SAIL Databank. We created two population-based cohorts derived from multiple demographic and healthcare data sources (Figure 1):

- The C20 cohort consists of all people alive and known to the National Health Service (NHS) in Wales from the 1st January 2020 with follow up until 31st May 2020. We include people who moved into, or were born in Wales after 1st January 2020. Follow-up data will be added prospectively and the C20 cohort will be updated on a monthly basis in line with a full month of coverage of available data. Linkage to other data sources is also available beyond the cohort end date where the cadence and quality of each data source allows its use for intelligence and analytics. Some datasets are analysed daily.
- The C16 cohort includes all individuals living in Wales and known to the NHS on the 1st January 2016 with follow up to the 31st December 2019. C16 is designed to provide counterfactual and contextual comparative population health service utilisation, and mortality rates.

Membership of both cohorts is based on the inclusion of a person's residence in Wales, registered to a Welsh General Practice, a free to use NHS system at the point of primary care registration in the UK, which is recorded within the Wales Demographic Service Dataset (WDSD). People are censored by study endpoint or migration out of Wales.

Data Sources

For peer review only

Baseline populations are created using the weekly updated WDSD, the monthly updated Office for National Statistics (ONS) mortality registry data known as the Annual District Death Extract (ADDE), two new COVID-19 daily data sources: the Consolidated Death Data Source (CDDS) created by NHS Wales Informatics Service (NWIS), and the Annual District Death Daily (ADDD) from ONS.

Anonymised Linkage Fields

Linkage fields are used to anonymously link between data sources in the SAIL Databank. SAIL utilises a multiple encryption system in which a trusted third party, the NHS Wales Informatics Service, uniquely matches identities to an Anonymised Linkage Field (ALF) and residences to a Residential Anonymised Linkage Field before uploading data to SAIL.[2,3,14]

Demographic data

The cohorts include the following variables: Anonymised Linkage Field (ALF), age, sex, date of death, date of movement out of Wales, Residential Anonymised Linkage Field (RALF) and Care Home Anonymised Linkage Fields (CHALFs) for older people at cohort inception. The CHALF was derived from a data extract from Care Inspectorate Wales in 2020 for all adult care home settings.[8] Geographical variables associated with the RALF and CHALF include Lower Layer Super Output Area (LSOA) 2011, which has been mapped to the Welsh Index of Multiple Deprivation (WIMD) version 2019 to derive deprivation quintiles; Welsh health board of residence; and urban/rurality categories.[15,16] Using Welsh Government's Pupil Level annual school census (PLASC), the school population can also be linked to the cohorts for analyses by school network.[17]

In addition, permission has been granted to embed occupation and role categories from electronic staff records of all NHS health boards and trusts, local authority social care workers and education staff. For healthcare workers, the electronic staff records system used in all health boards and trusts (111,000) are categorised by whether roles involve direct patient care or not and by occupational groups: Additional Professional Scientific and Technical; Additional Clinical Service; Administrative and Clerical; Allied Health Professionals; Estates and Ancillary; Healthcare Scientist; Medical and Dental, Nursing and Midwifery Registered; and Students. Social care workers are registered (https://socialcare.wales/) and grouped into

For peer review only

social workers, child home workers and domiciliary care workers (estimated 32,000). Educational staff records (estimated 70,000) include categories for teachers, support and administrative staff. This information is collected from the annual School Workforce Annual Census (SWAC) held by Welsh Government.[18] Data on care home staff is collected as part of Public Health Wales testing of all staff and residents and made available in SAIL through the standard Laboratory Information Management System (LIMS) dataset. Permission has also been granted to link 2011 ONS census fields on ethnicity, occupation, housing tenure, over-crowding and socio-economic status. Ethnicity codes are derived from multiple health and social data sources mapped to Census 2011 groupings.[19]

Health data

All hospital admissions, outpatient and emergency department attendances treated in NHS hospitals as well as GP data on all diagnoses and treatments from SAIL providing practices (80% population coverage) are available for cohort participants.[20] As of the beginning of 2020, we have added:

- Daily GP respiratory and COVID codes for 100% of Welsh GP practices.
- Daily COVID-19 antigen test results.
- Bi-weekly data on participants reporting symptoms through the KCL/ZOE symptom tracking app. [21]
- Weekly critical care data from the Intensive Care National Audit and Research Centre (ICNARC). [22-23]
- Bi-weekly COVID-19 viral genomic variant call format (VCF) data and viral lineage assignments from Public Health Wales (PHW).[24]
- Monthly community dispensing data from pharmacies providing NHS issued prescriptions, backdated to 2016.[25]

Exposure variables and potential confounding factors

A number of exposure variables will be used to contextualise the study primary outcomes, including age, sex, socioeconomic status (SES) and clinical risk groups.

Socio-economic status (SES) will be derived from WIMD with quintile 1 being the most deprived, and also at individual/household level from 2011 census using the following codes:

For peer review only

approximated social grade (SCGPUK11); highest level of qualification (HLQPUK11), and National Statistics Socio-economic Classification (NSSEC).[26]

Clinical risk groups have been derived from those used in scientific papers on predictors of influenza and COVID-19 outcomes,[27-28] published phenotype libraries including the 308 phenotypes created by the CALIBER study,[29] commonly used comorbidity indices (Charlson and Elixhauser),[30-31] and frailty indices (electronic Frailty Index for GP data and Hospital Frailty Risk Score).[32-34] In order to compare and combine results with other studies we will replicate the 19 clinical groups included in a similar study in Scotland.[35]

Microbiological testing data will be de-duplicated and used to generate case-data for standard case definitions, agreed at UK level where possible.

Body mass index (BMI) will be categorised as < 20, 20-24, 25-29, 30-39, ≥ 40 kg/m^2; and smoking status categorised into four groups: current smoker, non-smoker, ex-smoker and not recorded for patients with no data on smoking, replicated from a study carried out in Scotland.[35]

Statistical analysis

We will describe baseline characteristics for exposures and outcomes of interest utilising means, medians, proportions, odds ratios (ORs) and rate ratios (RRs) with appropriate measures of dispersion. We will report on prevalence of missing data by variable and utilise two tailed hypothesis tests with 5% significance level.

Non-independence of observations measured over time or within associated clusters, e.g. General Practice or households will be taken into account using random effects. We will use causal frameworks where causal relationships are implied.[36] Hypotheses being tested will be stated in advance and plans will be drawn up for each research project. Analyses will primarily be conducted in R statistical programming language.[37-38]

Analyses

For peer review only

We will test associations for demographic, socioeconomic, and clinical risk factors for COVID-19 infection and associated morbidity and mortality. COVID-19 infection will be defined in a number of ways: a) positive SARS-CoV-2 laboratory antigen test, b) clinical diagnosis of COVID-19 infection in GP records, intensive care, or hospital discharge records, c) ONS mortality records listing COVID19 as the underlying or contributory cause, and d) positive serology report (when available).

Planned analyses include:

- Incidence of COVID-19 over time and by geography and demographic groups.
- Influence of area deprivation and individual SES metrics on infection and outcomes.
- Impact of COVID-19 on short term (<6 months) and longer-term population outcomes such as changes in health service utilisation and excess, overall and disease-specific mortality.
- Description of the extent of clustering of cases within all available residential, educational, occupational, and geographic units, thus providing signatures of spatial spread at defined levels, and between levels, thought to have played a crucial role in transmission.

We will investigate the relationship between health (physical and mental), socioeconomic, and environmental factors, such as self-rated health, limiting long-term illness, housing tenure, over-crowding, education status, and occupation on infection risk and outcome.

Changes in healthcare utilisation will be assessed by measuring differences pre and post-infection for: NHS111 telephone calls, GP consultations, Emergency Department attendances, hospital admissions and length of stay, and intensive care admissions.

Analytical techniques will include descriptive statistics, univariate and multivariate generalised linear mixed models, survival analyses, and the use of self-controlled case series for temporary risk factors.[39] Relationships between variables will be clarified before specific analyses.

Cohort Characteristics

For peer review only - http://bmjopen.bmj.com/site/about/guidelines.xhtml

The C16 and C20 cohorts have been constructed from patients registered with all General Practice in Wales (Table 1).

Table 1: C16 and C20 cohort demographics to end May 2020.

**Cohort**	**C16**	**C20**
Individuals (N)	3,087,032	3,277,114
Cohort start date	2016-01-01	2020-01-01
Cohort end date	2019-12-31	2020-05-31
Deaths in period	117,565 (3.8%)	16,380 (0.5%)
Full coverage *(cohort end date = 2019-12-31/2020-05-31)*	2,651,957 (85.9%)	3,237,389 (98.8%)
Registered with a SAIL providing practice *(registration end date > cohort start date)*	2,608,761 (84.5%)	2,666,331 (81.4%)
Mean age (sd)	41.3 (23.7)	41.9 (23.8)
Sex		
Female	50.1%	50.1%
*WIMD 2019 Quintile		
1	20.3%	19.1%
2	19.9%	18.5%
3	20.1%	18.4%
4	19.7%	18.1%
5	19.9%	18.3%
Missing WIMD	0.0%	7.7%
**WIMD 2019 Quintile: 1 = most deprived, 5 = least deprived, please note a one decimal place rounding error.*		

Power to detect relevant outcomes will be assessed as the pandemic evolves. There are plans to collaborate with researchers across the UK and collating data from similar cohorts,[35] to maximise power, support the evaluation of natural experiments in policy and timing around disease control, and exit strategies from the lockdown on physical restrictions that commenced on March 23rd 2020.

The individual datasets that comprise the cohorts are held on the globally accessible SAIL databank available to accredited researchers.

Proposed future developments

For peer review only

As the pandemic evolves so will policies and practices to control the epidemic and mitigate negative consequences. As these develop, we plan to utilise the cohorts as a platform for their evaluation, by linking dates and presence of interventions as data become available. There are subtle differences in the timing and approaches to controlling the epidemic in diverse settings and in exiting lockdown across the four UK nations. This provides opportunities for collaborative and timely evaluation of natural experiments of policies and approaches across the UK, which would refine evidence-based exit strategies.

Patient and public involvement

This study is based on an extension of the developing Wales Multi-morbidity Cohort (WMC). CD and JD are members of the public were involved in the design of the WMC and C20/C16 studies. Additional members of the public are in the process of being recruited to the research steering committee to represent the views of health, social care and educational staff.

Ethics and dissemination

SAIL's independent Information Governance Review Panel (IGRP),[5] has approved a submission to allow the use of WMC with additional data flows to aid the COVID-19 research response (SAIL project 0911). IGRP applications are scrutinised by members of the public; only those applications that can demonstrate privacy protection and are in the public interest are approved. SAIL's Consumer Panel, comprising members of the public, were consulted during the development of WMC. Two members of the public were recruited to the study steering group following approval.

Contributors

All authors contributed to the conception and or design of aspects of the study. JL is the lead analyst for the Wales Multi-morbidity Cohort creation and designed the data framework for the C20/C16 cohorts. JL, AA, FT, GD, LN, RG, RB, JH, RF, ST, DT, JR, AM, CO, SET, LA, TS, DT, CE, TC, CT, RP, GJ, SS, JH, AMC created meta-data, prepared or linked datasets to create the cohort. JL, AA, FT, GD, LN, RG, RB, JH, RF, ST, DT, JR, AM, CO, SET, LA, LC, MG, SB, BL, AJ, TS, JD, CD, DRhT, CW, CE, SC, TC, CT, RP, PD, GJ, SS, JH, AMC, KH, RAL contributed to the drafting

of the manuscript and gave final approval of the version to be published. RAL is the principle investigator and guarantor of the study.

Acknowledgements

The authors would like to acknowledge that this work uses data provided by patients and collected by the NHS as part of their care and support and the Understanding Patient Data initiative.[40] We would also like to acknowledge all data providers who make anonymised data available for research.

We wish to acknowledge the collaborative partnership that enabled acquisition and access to the de-identified data, which led to this output. The collaboration was led by the Swansea University Health Data Research UK team under the direction of the Welsh Government Technical Advisory Group (TAG), and includes the following groups and organisations: the Secure Anonymised Information Linkage (SAIL) Databank, Administrative Data Research (ADR) Wales, NHS Wales Informatics Service (NWIS), Public Health Wales, NHS Shared Services and the Welsh Ambulance Service Trust (WAST). All research conducted has been completed under the permission and approval of the SAIL independent Information Governance Review Panel (IGRP) project number 0911.

Funding

The WMC, on which components of this study are based, is funded by Health Data Research UK and the Medical Research Council (MR/S027750/1). Health Data Research UK is funded by the UK Medical Research Council, Engineering and Physical Sciences Research Council, Economic and Social Research Council, National Institute for Health Research (England), Chief Scientist Office of the Scottish Government Health and Social Care Directorates, Health and Social Care Research and Development Division (Welsh Government), Public Health Agency (Northern Ireland), British Heart Foundation and Wellcome.

Disclaimer

The views and opinions expressed therein are those of the authors and do not necessarily reflect those of the funding agencies, NHS organisations or Welsh Government.

For peer review only

Competing interests

None declared.

Provenance and peer review

The WMC was peer–reviewed and specific objectives funded by the Medical Research Council (MR/S027750/1). This COVID19 extension has been peer-reviewed and an award will be made by a major UK research funder. Details will be included as soon as the funder makes an official announcement.

For peer review only

References

1. COVID-19 Dashboard by the Centre for Systems Science and Engineering (CSSE) at Johns Hopkins University (JHU). Available: https://coronavirus.jhu.edu/map.html [Accessed 1 June 2020].
2. Lyons R, Jones K, John G, Brooks C, Verplancke J, Ford D et al. The SAIL databank: linking multiple health and social care datasets. *BMC Medical Informatics and Decision Making* 2009;9(1).
3. Ford DV, Jones KH, Verplancke J-P, Lyons RA, John G, Brown G, et al. The SAIL Databank: building a national architecture for e-health research and evaluation. *BMC Health Services Research* 2009;9(1).
4. Lyons RA, Ford DV, Moore L, Rodgers SE. Use of data linkage to measure the population health effect of non-health-care interventions. The Lancet. 2014;383(9927):1517–9.
5. Jones KH, Ford DV, Jones C, Dsilva R, Thompson S, Brooks CJ, et al. A case study of the Secure Anonymous Information Linkage (SAIL) Gateway: A privacy-protecting remote access system for health-related research and evaluation. Journal of Biomedical Informatics. 2014;50:196–204.
6. Lyons RA, Turner S, Lyons J, Walters A, Snooks HA, Greenacre J, et al. All Wales Injury Surveillance System revised: development of a population-based system to evaluate single-level and multilevel interventions. Injury Prevention. 2015;22(Suppl 1):i50–i55.
7. Snooks HA, Anthony R, Chatters R, Dale J, Fothergill R, Gaze S, et al. Support and Assessment for Fall Emergency Referrals (SAFER) 2: a cluster randomised trial and systematic review of clinical effectiveness and cost-effectiveness of new protocols for emergency ambulance paramedics to assess older people following a fall with referral to community-based care when appropriate. Health Technology Assessment. 2017;21(13):1–218.
8. Hollinghurst J, Akbari A, Fry R, Watkins A, Berridge D, Clegg A, et al. Study protocol for investigating the impact of community home modification services on hospital utilisation for fall injuries: a controlled longitudinal study using data linkage. BMJ Open. 2018;8(10).
9. Mizen A, Song J, Fry R, Akbari A, Berridge D, Parker SC, et al. Longitudinal access and exposure to green-blue spaces and individual-level mental health and well-being: protocol for a longitudinal, population-wide record-linked natural experiment. BMJ Open. 2019;9(4).

For peer review only

10. Szakmany T, Walters AM, Pugh R, Battle C, Berridge DM, Lyons RA. Risk Factors for 1-Year Mortality and Hospital Utilization Patterns in Critical Care Survivors. Critical Care Medicine. 2019;47(1):15–22.
11. Rodgers SE, Bailey R, Johnson R, Berridge D, Poortinga W, Lannon S, et al. Emergency hospital admissions associated with a non-randomised housing intervention meeting national housing quality standards: a longitudinal data linkage study. Journal of Epidemiology and Community Health. 2018;72(10):896–903.
12. Paranjothy S, Evans A, Bandyopadhyay A, Fone D, Schofield B, John A, et al. Risk of emergency hospital admission in children associated with mental disorders and alcohol misuse in the household: an electronic birth cohort study. The Lancet Public Health. 2018;3(6).
13. Schnier C, Wilkinson T, Akbari A, Orton C, Sleegers K, Gallacher J, et al. Cohort profile: The Secure Anonymised Information Linkage databank Dementia e-cohort (SAIL-DeC). International Journal of Population Data Science. 2020;5(1).
14. Rodgers SE, Lyons RA, Dsilva R, Jones KH, Brooks CJ, Ford DV, et al. Residential Anonymous Linking Fields (RALFs): a novel information infrastructure to study the interaction between the environment and individuals' health. Journal of Public Health. 2009;31(4):582–8.
15. Welsh Index Multiple Deprivation Index. Available: https://gov.wales/welsh-index-multiple-deprivation-index-guidance [Accessed 9 April 2020].
16. 2011 rural/urban classifications. Available: https://www.ons.gov.uk/methodology/geography/geographicalproducts/ruralurbanclassifications/2011ruralurbanclassification [Accessed 9 April 2020].
17. Welsh Government. Pupil level annual school census (PLASC). Available: https://gov.wales/pupil-level-annual-school-census-plasc [Accessed 14 June 2020].
18. Welsh Government. School Workforce Annual Census. Welsh Government. Available: https://gov.wales/school-workforce-annual-census- [Accessed 16 June 2020].
19. Office of National Statistics. 2011 Census analysis: ethnicity and religion of the non-UK born population in England and Wales: 2011. Available: https://www.ons.gov.uk/peoplepopulationandcommunity/culturalidentity/ethnicity/articles/2011censusanalysisethnicityandreligionofthenonukbornpopulationinenglandandwales/2015-06-18 [Accessed 16 June 2020].
20. Thayer D, Rees A, Kennedy J, Collins H, Harris D, Halcox J, et al. Measuring follow-up time in routinely-collected health datasets: Challenges and solutions. Plos One. 2020;15(2).

For peer review only

21. Menni, C., Valdes, A.M., Freidin, M.B. *et al.* Real-time tracking of self-reported symptoms to predict potential COVID-19. *Nat Med* (2020). https://doi.org/10.1038/s41591-020-0916-2
22. Intensive Care National Audit and Research Centre (ICNARC). ICNARC report on COVID-19 in critical care in Wales. *Available:* https://www.icnarc.org/Our-Audit/Audits/Cmp/Reports *[Accessed* 02 July 2020*].*
23. Intensive Care National Audit and Research Centre (ICNARC). ICNARC report on COVID-19 in critical care 26 June 2020*. Available:* https://www.icnarc.org/Our-Audit/Audits/Cmp/Reports *[Accessed* 02 July 2020*].*
24. An integrated national scale SARS-CoV-2 genomic surveillance network. The Lancet Microbe. 2020;1(3).
25. NHS Wales Shared Services. Community pharmacy dispensing data. Available: https://nwssp.nhs.wales/ourservices/primary-care-services/general-information/data-and-publications/pharmacy-practice-dispensing-data/ [Accessed 24 June 2020].
26. Office for National Statistics. Ethnicity, disability, religion. Which groups are most at risk of death involving COVID19? Available: https://blog.ons.gov.uk/2020/06/19/ethnicity-disability-religion-which-groups-are-at-most-risk-of-death-involving-covid-19/ [Accessed 24 June 2020].
27. Harrison EM, Docherty AB, Barr B, Buchan I, Carson G, Drake TM, et al. Ethnicity and Outcomes from COVID-19: The ISARIC CCP-UK Prospective Observational Cohort Study of Hospitalised Patients. SSRN Electronic Journal. 2020;
28. Docherty AB, Harrison EM, Green CA, Hardwick HE, Pius R, Norman L, et al. Features of 20 133 UK patients in hospital with covid-19 using the ISARIC WHO Clinical Characterisation Protocol: prospective observational cohort study. Bmj. 2020;:m1985.
29. Kuan V, Denaxas S, Gonzalez-Izquierdo A, Direk K, Bhatti O, Husain S, et al. A chronological map of 308 physical and mental health conditions from 4 million individuals in the English National Health Service. The Lancet Digital Health. 2019;1(2).
30. Charlson ME, Pompei P, Ales KL, Mackenzie C. A new method of classifying prognostic comorbidity in longitudinal studies: Development and validation. Journal of Chronic Diseases. 1987;40(5):373–83.
31. Thompson NR, Fan Y, Dalton JE, Jehi L, Rosenbaum BP, Vadera S, et al. A New Elixhauser-based Comorbidity Summary Measure to Predict In-Hospital Mortality. Medical Care. 2015;53(4):374–9.

For peer review only

32. Hollinghurst J, Fry R, Akbari A, Clegg A, Lyons RA, Watkins A, et al. External validation of the electronic Frailty Index using the population of Wales within the Secure Anonymised Information Linkage Databank. Age and Ageing. 2019;48(6):922–6.
33. Gilbert T, Neuburger J, Kraindler J, Keeble E, Smith P, Ariti C, et al. Development and validation of a Hospital Frailty Risk Score focusing on older people in acute care settings using electronic hospital records: an observational study. The Lancet. 2018;391(10132):1775–82.
34. Clegg A, Bates C, Young J, Ryan R, Nichols L, Teale EA, et al. Development and validation of an electronic frailty index using routine primary care electronic health record data. Age and Ageing. 2017;
35. Simpson CR, Robertson C, Vasileiou E, Mcmenamin J, Gunson R, Ritchie LD, et al. Early Pandemic Evaluation and Enhanced Surveillance of COVID-19 (EAVE II): protocol for an observational study using linked Scottish national data. BMJ Open. 2020;10(6).
36. Victora CG, Huttly SR, Fuchs SC, Olinto MT. The role of conceptual frameworks in epidemiological analysis: a hierarchical approach. International Journal of Epidemiology. 1997;26(1):224–7.
37. R Studio Team (2020). RStudio: Integrated Development for R. RStudio, PBC, Boston, MA URL. Available: http://www.rstudio.com/.
38. R Core Team (2019). R: A language and environment for statistical computing. R foundation for Statistical Computing, Vienna, Austria. Available: http://www.R-project.org/.
39. Petersen I, Douglas I, Whitaker H. Self controlled case series methods: an alternative to standard epidemiological study designs. Bmj. 2016;:i4515.
40. Understanding patient Data. Available: https://understandingpatientdata.org.uk/supporting-conversations [Accessed 23 June 2020].

For peer review only

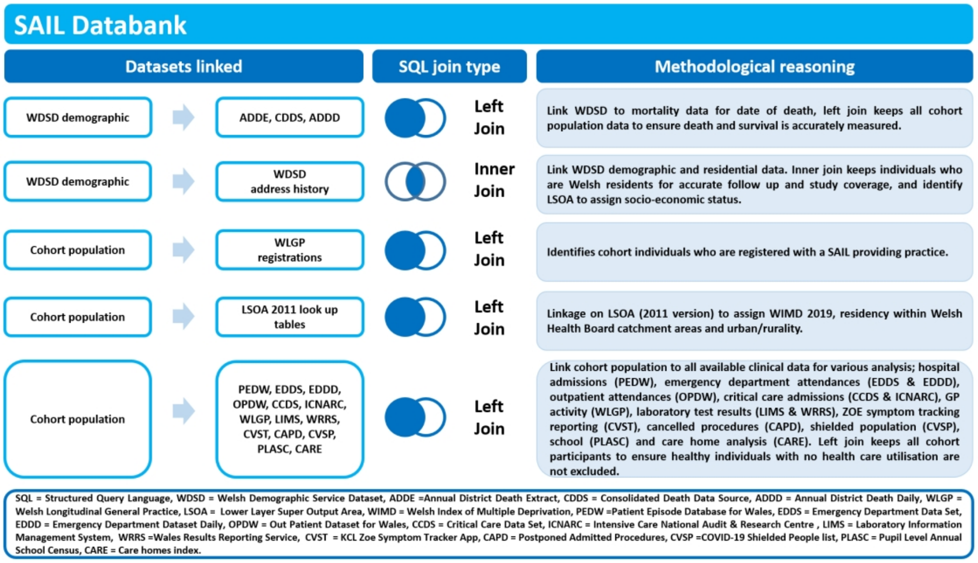

Figure 1: Data linkage of multiple demographic and healthcare data sources used in the creation of two population wide cohorts: C20 and C16.

342x194mm (150 x 150 DPI)

BMJ Open

Understanding and responding to COVID-19 in Wales: protocol for a privacy protecting data platform for enhanced epidemiology and evaluation of interventions.

Journal:	*BMJ Open*
Manuscript ID	bmjopen-2020-043010.R1
Article Type:	Protocol
Date Submitted by the Author:	21-Sep-2020
Complete List of Authors:	Lyons, Jane; Swansea University Medical School, Population Data Science<br>Akbari, Ashley; Swansea University Medical School, Population Data Science<br>Torabi, Fatemeh; Swansea University Medical School, Population Data Science<br>Davies, Gareth; Swansea University Medical School, Population Data Science<br>North, Laura; Swansea University Medical School, Population Data Science<br>Griffiths, Rowena; Swansea University Medical School, Population Data Science<br>Bailey, Rowena; Swansea University Medical School, Population Data Science<br>Hollinghurst, Joseph; Swansea University Medical School, Population Data Science<br>Fry, Richard; Swansea University Medical School, Population Data Science<br>Turner, Samantha L.; Swansea University Medical School, Population Data Science<br>Thompson, Daniel; Swansea University Medical School, Population Data Science<br>Rafferty, James; Swansea University Medical School, Population Data Science<br>Mizen, Amy; Swansea University Medical School, Population Data Science<br>Orton, Chris; Swansea University Medical School, Population Data Science<br>Thompson, Simon; Swansea University Medical School, Population Data Science<br>Au-Yeung, Lee; Swansea University Medical School, Population Data Science<br>Cross, Lynsey; Swansea University Medical School, Population Data Science<br>Gravenor, Mike; Swansea University Medical School, School of Medicine<br>Brophy, Sinead; Swansea University Medical School, Population Data Science<br>Lucini, Biagio; Swansea University Medical School, Population Data Science

	John, Ann; Swansea University Medical School, Population Data Science<br>Szakmany, Tamas; Cardiff University, Department of Anaesthesia, Intensive Care and Pain Medicine, Division of Population Medicine; Aneurin Bevan University Health Board, Critical Care Directorate<br>Davies, Jan<br>Davies, Chris<br>Thomas, Daniel; Public Health Wales<br>Williams, Christopher; Public Health Wales<br>Emmerson, Chris; Public Health Wales<br>Cottrell, Simon; Public Health Wales<br>Connor, Thomas; Cardiff University, School of Biosciences<br>Taylor, Chris; Cardiff University<br>Pugh, Richard; Glan Clwyd Hospital<br>Diggle, Peter; Lancaster University, Faculty of Health and Medicine; University of Liverpool, Epidemiology and Population Health<br>John, Gareth; NHS Wales Informatics Service,<br>Scourfield, Simon; NHS Wales Information Service<br>Hunt, Joe; NHS Wales Information Service<br>Cunningham, Anne Marie; NHS Wales Information Service<br>Helliwell, Kathryn; Welsh Government<br>Lyons, Ronan; Swansea University Medical School, Population Data Science
**Primary Subject Heading**:	Public health
Secondary Subject Heading:	Epidemiology, Health informatics, Health services research
Keywords:	COVID-19, EPIDEMIOLOGY, PUBLIC HEALTH, Health informatics < BIOTECHNOLOGY & BIOINFORMATICS

SCHOLARONE™
Manuscripts

I, the Submitting Author has the right to grant and does grant on behalf of all authors of the Work (as defined in the below author licence), an exclusive licence and/or a non-exclusive licence for contributions from authors who are: i) UK Crown employees; ii) where BMJ has agreed a CC-BY licence shall apply, and/or iii) in accordance with the terms applicable for US Federal Government officers or employees acting as part of their official duties; on a worldwide, perpetual, irrevocable, royalty-free basis to BMJ Publishing Group Ltd ("BMJ") its licensees and where the relevant Journal is co-owned by BMJ to the co-owners of the Journal, to publish the Work in this journal and any other BMJ products and to exploit all rights, as set out in our licence.

The Submitting Author accepts and understands that any supply made under these terms is made by BMJ to the Submitting Author unless you are acting as an employee on behalf of your employer or a postgraduate student of an affiliated institution which is paying any applicable article publishing charge ("APC") for Open Access articles. Where the Submitting Author wishes to make the Work available on an Open Access basis (and intends to pay the relevant APC), the terms of reuse of such Open Access shall be governed by a Creative Commons licence – details of these licences and which Creative Commons licence will apply to this Work are set out in our licence referred to above.

Other than as permitted in any relevant BMJ Author's Self Archiving Policies, I confirm this Work has not been accepted for publication elsewhere, is not being considered for publication elsewhere and does not duplicate material already published. I confirm all authors consent to publication of this Work and authorise the granting of this licence.

For peer review only

Understanding and responding to COVID-19 in Wales: protocol for a privacy protecting data platform for enhanced epidemiology and evaluation of interventions.

Jane Lyons, Data Science Building, Population Data Science, Swansea University Medical School, Singleton Park, Swansea, SA2 8PP, UK, J.Lyons@Swansea.ac.uk, 01792 513028 (corresponding author)

Ashley Akbari, Population Data Science, Swansea University Medical School, Swansea, UK

Fatemeh Torabi, Population Data Science, Swansea University Medical School, Swansea, UK

Gareth Davies, Population Data Science, Swansea University Medical School, Swansea, UK

Dr Laura North, Population Data Science, Swansea University Medical School, Swansea, UK

Rowena Griffiths, Population Data Science, Swansea University Medical School, Swansea, UK

Rowena Bailey, Population Data Science, Swansea University Medical School, Swansea, UK

Dr Joseph Hollinghurst, Population Data Science, Swansea University Medical School, Swansea, UK

Dr Richard Fry, Population Data Science, Swansea University Medical School, Swansea, UK

Samantha Turner, Population Data Science, Swansea University Medical School, Swansea, UK

Dr Daniel Thompson, Population Data Science, Swansea University Medical School, Swansea, UK

Dr James Rafferty, Population Data Science, Swansea University Medical School, Swansea, UK

Dr Amy Mizen, Population Data Science, Swansea University Medical School, Swansea, UK

Chris Orton, Population Data Science, Swansea University Medical School, Swansea, UK

Simon Thompson, Population Data Science, Swansea University Medical School, Swansea, UK

Lee Au-Yeung, Population Data Science, Swansea University Medical School, Swansea, UK

Lynsey Cross, Population Data Science, Swansea University Medical School, Swansea, UK

Professor Mike Gravenor, Population Data Science, Swansea University Medical School, Swansea, UK

Professor Sinead Brophy, Population Data Science, Swansea University Medical School, Swansea, UK

Professor Biagio Lucini, Population Data Science, Swansea University Medical School, Swansea, UK

For peer review only

Professor Ann John, Population Data Science, Swansea University Medical School, Swansea, UK

Professor Tamas Szakmany, Cardiff University, Cardiff, UK

Jan Davies, Member of public, Swansea, UK

Chris Davies, Member of public, Swansea, UK

Professor Daniel Rh Thomas, Public Health Wales, Cardiff, UK

Dr Christopher Williams, Public Health Wales, Cardiff, UK

Chris Emmerson, Public Health Wales, Cardiff, UK

Dr Simon Cottrell, Public Health Wales, Cardiff, UK

Dr Thomas Connor, Cardiff University, Cardiff, UK

Professor Chris Taylor, Cardiff University, Cardiff, UK

Dr Richard Pugh, Betsi Cadwaladr University Health Board, Rhyl, UK

Prof Peter J Diggle, Lancaster University, Lancaster, UK

Dr Gareth John, NHS Wales Informatics Service, Cardiff, UK

Simon Scourfield, NHS Wales Informatics Service, Cardiff, UK

Joe Hunt, NHS Wales Informatics Service, Cardiff, UK

Dr Anne Marie Cunningham, NHS Wales Informatics Service, Cardiff, UK

Kathryn Helliwell, Welsh Government, Cardiff, UK

Professor Ronan A Lyons, Population Data Science, Swansea University Medical School, Swansea, UK

Keywords: COVID-19, Data linkage, epidemiology, public health, evaluation

Word count: 2,217

For peer review only

ABSTRACT

Introduction

The emergence of the novel respiratory SARS-CoV-2 and subsequent COVID-19 pandemic has required rapid assimilation of population-level data to understand and control the spread of infection in the general and vulnerable populations. Rapid analyses are needed to inform policy development and target interventions to at risk groups to prevent serious health outcomes. We aim to provide an accessible research platform to determine demographic, socioeconomic and clinical risk factors for infection, morbidity, and mortality of COVID-19, to measure the impact of COVID-19 on healthcare utilisation and long-term health, and to enable the evaluation of natural experiments of policy interventions.

Methods and analysis

Two privacy-protecting population-level cohorts have been created and derived from multi-sourced demographic and healthcare data. The C20 cohort consists of 3.2 million people in Wales on the 1st January 2020 with follow up until 31st May 2020. The complete cohort dataset will be updated monthly with some individual datasets available daily. The C16 cohort consists of 3 million people in Wales on the 1st January 2016 with follow up to the 31st December 2019. C16 is designed as a counterfactual cohort to provide contextual comparative population data on disease, health service utilisation, and mortality. Study outcomes will: a) characterise the epidemiology of COVID-19, b) assess socioeconomic and demographic influences on infection and outcomes, c) measure impact of COVID-19 on short term and longer-term population outcomes and d) undertake studies on the transmission and spatial spread of infection.

Ethics and dissemination

The Secure Anonymised Information Linkage (SAIL) independent Information Governance Review Panel (IGPR) has approved this study. The study findings will be presented to policy groups, public meetings, national and international conferences, and published in in peer-reviewed journals.

For peer review only

Strengths and limitations of this study

- Rapid access to multiple data sources on a complete population.
- Great variety of individual and household level data on demography, disease status, morbidity, mortality and viral genomics to support a wide range of studies on the evolution of the epidemic in Wales.
- Ability to support hierarchical analyses at varying geographical units: private residences, care homes, educational setting and healthcare facilities to examine spatial spread and transmission of SARS-CoV-2 to inform and evaluate targeting of interventions.
- However, routine data does not capture data on some important aspects, such as quality of life.

For peer review only

INTRODUCTION

Understanding and controlling the COVID-19 pandemic is a rapidly changing, complex issue that requires near real-time local data, analyses, modelling and multidisciplinary team science to devise, implement and evaluate a wide variety of inter- and cross-sectoral interventions to minimise population harm.[1]

As the pandemic evolves a wide range of issues need to be considered including: the spread of infection in the general and vulnerable populations; health service resilience; indirect harm minimisation; and effectiveness of control policies and interventions.

Responding to this challenge, the Welsh Government created a COVID-19 Technical Advisory Group (TAG) to provide rapid assimilation of available evidence and guide analysis of data to inform policy development and appraisal. Insight from linked data is seen as being essential to understand the evolving epidemic. TAG commissioned the support of analyses conducted through the Secure Anonymised Information Linkage (SAIL) Databank (www.saildatabank.com) to formulate evidence and advice to underpin its work in responding to COVID-19.[2-5] SAIL is a state of the art, remotely accessible, privacy-protecting system, accredited under the Digital Economy Act. SAIL holds and provides access to linked de-identified data from multiple sources at individual, household and multiple ecological levels, for the population of Wales. The SAIL Databank has previously supported numerous types of clinical and population studies, including cohorts, evaluations of natural experiments and embedded trials.[6-13]

For peer review only

This paper describes the development of two population-based cohorts in Wales, derived from multiple data sources to provide near real-time, in-pandemic intelligence and analytics to TAG in relation to the following broad objectives:

Primary objectives

a) Determine demographic, socioeconomic and clinical risk factors for infection, morbidity, and mortality related COVID-19;
b) Determine risk of COVID-19 infection and outcomes in occupational groups; and
c) Measure the population impact of COVID-19 on healthcare utilisation.

Secondary objectives

a) Create a platform to enable the evaluation of policies and interventions aimed at controlling the epidemic, whether clinical or non-pharmaceutical in nature; and
b) Provide access to these derived population-based cohorts and linked data sources to organisation and people with relevant skills and expertise within the NHS, academia and government.

METHODS

Study design and population

The cohorts were derived from de-identified linked data from the SAIL Databank. We created two population-based cohorts derived from multiple demographic and healthcare data sources (Figure 1):

- The C20 cohort consists of all people alive and known to the National Health Service (NHS) in Wales from the 1st January 2020 with follow up until 31st May 2020. We include people who moved into or were born in Wales after 1st January 2020. Follow-up data will be added prospectively and the C20 cohort will be updated on a monthly basis in line with a full month of coverage of available data. Linkage to other data sources is also available beyond the cohort end date where the frequency and quality of each data source allows its use. Some datasets are analysed daily.
- The C16 cohort includes all individuals living in Wales and known to the NHS on the 1st January 2016 with follow up to the 31st December 2019. C16 is designed to provide counterfactual and contextual comparative data on population health service utilisation, and mortality rates.

Membership of both cohorts is based on the inclusion of a person's residence in Wales, registered to a Welsh General Practice, a free to use NHS system at the point of primary care registration in the UK (Figure 2). This is recorded within the Wales Demographic Service Dataset (WDSD). People are censored by study endpoint or migration out of Wales.

Data Sources

For peer review only

Baseline populations are created using the weekly updated WDSD, the monthly updated Office for National Statistics (ONS) mortality registry data known as the Annual District Death Extract (ADDE), two new COVID-19 daily data sources: the Consolidated Death Data Source (CDDS) created by NHS Wales Informatics Service (NWIS), and the Annual District Death Daily (ADDD) from ONS.

Anonymised Linkage Fields

Linkage fields are used to anonymously link between data sources in the SAIL Databank. SAIL utilises a multiple encryption system in which a trusted third party, the NHS Wales Informatics Service, uniquely matches identities to an Anonymised Linkage Field (ALF) and residences to a Residential Anonymised Linkage Field before uploading data to SAIL.[2,3,14]

Demographic data

The cohorts include the following variables: Anonymised Linkage Field (ALF), age, sex, date of death, date of movement out of Wales, Residential Anonymised Linkage Field (RALF) and Care Home Anonymised Linkage Fields (CHALFs) for older people at cohort inception. The CHALF was derived from a data extract from Care Inspectorate Wales in 2020 for all adult care home settings.[8] Geographical variables associated with the RALF and CHALF include Lower layer Super Output Area (LSOA) 2011 boundaries which are small statistical areas containing around 1500 people. LSOA 2011 has been mapped to the Welsh Index of Multiple Deprivation (WIMD) version 2019 to derive deprivation quintiles; Welsh health board of residence; and urban/rurality categories.[15,16] Using Welsh Government's Pupil Level annual school census (PLASC), the school population can also be linked to the cohorts for analyses by school network.[17]

In addition, permission has been granted to embed occupation and role categories from electronic staff records of all NHS health boards and trusts, local authority social care workers and education staff. For healthcare workers, the electronic staff records system used in all health boards and trusts (111,000) are categorised by whether roles involve direct patient care or not and by occupational groups: Additional Professional Scientific and Technical; Additional Clinical Service; Administrative and Clerical; Allied Health Professionals; Estates and Ancillary; Healthcare Scientist; Medical and Dental, Nursing and Midwifery Registered;

For peer review only

and Students. Social care workers are registered (https://socialcare.wales/) and grouped into social workers, child home workers and domiciliary care workers (estimated 32,000). Educational staff records (estimated 70,000) include categories for teachers, support and administrative staff. This information is collected from the annual School Workforce Annual Census (SWAC) held by Welsh Government.[18] Data on care home staff is collected as part of Public Health Wales testing of all staff and residents and made available in SAIL through the standard Laboratory Information Management System (LIMS) dataset. Permission has also been granted to link 2011 ONS census fields on ethnicity, occupation, housing tenure, over-crowding and socio-economic status. Ethnicity codes are derived from multiple health and social data sources mapped to Census 2011 groupings.[19]

Health data

All hospital admissions, outpatient and emergency department attendances treated in NHS hospitals as well as GP data on all diagnoses and treatments from SAIL providing practices (80% population coverage) are available for cohort participants.[20] As of the beginning of 2020, we have added:

- Daily GP respiratory and COVID codes for 100% of Welsh GP practices.
- Daily COVID-19 antigen test results.
- Bi-weekly data on participants reporting symptoms through the KCL/ZOE symptom tracking app.[21]
- Weekly critical care data from the Intensive Care National Audit and Research Centre (ICNARC).[22-23]
- Bi-weekly COVID-19 viral genomic variant call format (VCF) data and viral lineage assignments from Public Health Wales (PHW).[24]
- Monthly community dispensing data from pharmacies providing NHS issued prescriptions, backdated to 2016.[25]

Exposure variables and potential confounding factors

A number of exposure variables will be used to contextualise the study primary outcomes, including age, sex, socioeconomic status (SES) and clinical risk groups.

For peer review only

Socio-economic status (SES) will be derived from WIMD with quintile 1 including the 20% most deprived areas, and also at individual/household level from 2011 census using the following codes: approximated social grade (SCGPUK11); highest level of qualification (HLQPUK11), and National Statistics Socio-economic Classification (NSSEC).[26]

Clinical risk groups have been derived from those used in scientific papers:

- Predictors of influenza and COVID-19 outcomes.[27-28]
- Published phenotype (disease conditions) libraries including the 308 phenotypes created by the CALIBER study.[29]
- Commonly used comorbidity indices (Charlson and Elixhauser).[30-31]
- Frailty indices (electronic Frailty Index for GP data and Hospital Frailty Risk Score).[32-34]

In order to compare and combine results with other studies we will replicate the 19 clinical groups included in a similar study in Scotland.[35]

Microbiological testing data will be de-duplicated and used to generate case-data for standard case definitions, agreed at UK level where possible.

Body mass index (BMI) will be categorised as < 20, 20-24, 25-29, 30-39, ≥ 40 kg/m^2; and smoking status categorised into four groups: current smoker, non-smoker, ex-smoker and not recorded for patients with no data on smoking, replicated from a study carried out in Scotland.[35]

Statistical analysis

We will describe baseline characteristics for exposures and outcomes of interest utilising means, medians, proportions, odds ratios (ORs) and rate ratios (RRs) with appropriate measures of dispersion. We will report on prevalence of missing data by variable and utilise two tailed hypothesis tests with 5% significance level.

Non-independence of observations measured over time or within associated clusters, e.g. General Practice or households will be taken into account using random effects. We will use

For peer review only

causal frameworks where causal relationships are implied.[36] Hypotheses being tested will be stated in advance and plans will be drawn up for each research project. Analyses will primarily be conducted in R statistical programming language.[37-38]

Analyses

We will test associations for demographic, socioeconomic, and clinical risk factors for COVID-19 infection and associated morbidity and mortality. COVID-19 infection will be defined in a number of ways: a) positive SARS-CoV-2 laboratory antigen test, b) clinical diagnosis of COVID-19 infection in GP records, intensive care, or hospital discharge records, c) ONS mortality records listing COVID19 as the underlying or contributory cause, and d) positive serology report (when available).

Planned analyses include:

- Incidence of COVID-19 over time and by geography and demographic groups.
- Influence of area deprivation and individual SES metrics on infection and outcomes.
- Impact of COVID-19 on short term (<6 months) and longer-term population outcomes such as changes in health service utilisation and excess, overall and disease-specific mortality.
- Description of the extent of clustering of cases within all available residential, educational, occupational, and geographic units, thus providing signatures of spatial spread at defined levels, and between levels, thought to have played a crucial role in transmission.

We will investigate the relationship between health (physical and mental), socioeconomic, and environmental factors, such as self-rated health, limiting long-term illness, housing tenure, over-crowding, education status, and occupation on infection risk and outcome.

Changes in healthcare utilisation will be assessed by measuring differences pre and post-infection for: NHS111 telephone calls, GP consultations, emergency department attendances, hospital admissions and length of stay, and intensive care admissions.

For peer review only

Analytical techniques will include descriptive statistics, univariate and multivariate generalised linear mixed models, survival analyses, and the use of self-controlled case series for temporary risk factors.[39] Relationships between variables will be clarified before specific analyses.

Cohort Characteristics

The C16 and C20 cohorts have been constructed from patients registered with all General Practice in Wales (Table 1).

Table 1: C16 and C20 cohort demographics to end May 2020.

**Cohort**	**C16**	**C20**
Individuals (N)	3,087,032	3,277,114
Cohort start date	2016-01-01	2020-01-01
Cohort end date	2019-12-31	2020-05-31
Deaths in period	117,565 (3.8%)	16,380 (0.5%)
Full coverage *(cohort end date = 2019-12-31/2020-05-31)*	2,651,957 (85.9%)	3,237,389 (98.8%)
Registered with a SAIL providing practice *(registration end date > cohort start date)*	2,608,761 (84.5%)	2,666,331 (81.4%)
Mean age (sd)	41.3 (23.7)	41.9 (23.8)
Sex		
Female	50.1%	50.1%
*WIMD 2019 Quintile		
1	20.3%	19.1%
2	19.9%	18.5%
3	20.1%	18.4%
4	19.7%	18.1%
5	19.9%	18.3%
Missing WIMD	0.0%	7.7%
**WIMD 2019 Quintile: 1 = most deprived, 5 = least deprived, please note a one decimal place rounding error.*		

Power to detect relevant outcomes will be assessed as the pandemic evolves. There are plans to collaborate with researchers across the UK and collating data from similar cohorts,[35] to maximise power, support the evaluation of natural experiments in policy and its timing

around disease control and exit strategies from the lockdown on physical restrictions that commenced on March 23rd 2020.

The individual datasets that comprise the cohorts are held on the globally accessible SAIL databank available to accredited researchers.

Proposed future developments

As the pandemic evolves so will policies and practices to control the epidemic and mitigate negative consequences. As these develop, we plan to utilise the cohorts as a platform for their evaluation, by linking dates and presence of interventions as data become available. There are subtle differences in the timing and approaches to controlling the epidemic in diverse settings and in exiting lockdown across the four UK nations. This provides opportunities for collaborative and timely evaluation of natural experiments of policies and approaches across the UK, which would refine evidence-based exit strategies. We are also keen to contribute to international initiatives.

Patient and public involvement

This study is based on an extension of the developing Wales Multi-morbidity Cohort (WMC). CD and JD are members of the public were involved in the design of the WMC and C20/C16 studies. Additional members of the public are in the process of being recruited to the research steering committee to represent the views of health, social care and educational staff.

Ethics and dissemination

SAIL's independent Information Governance Review Panel (IGRP),[5] has approved a submission to allow the use of WMC with additional data flows to aid the COVID-19 research response (SAIL project 0911). IGRP applications are scrutinised by members of the public; only those applications that can demonstrate privacy protection and are in the public interest are approved. SAIL's Consumer Panel, comprising members of the public, were consulted during the development of WMC. Two members of the public were recruited to the study steering group following approval.

Contributors

For peer review only

All authors contributed to the conception and or design of aspects of the study. JL is the lead analyst for the Wales Multi-morbidity Cohort creation and designed the data framework for the C20/C16 cohorts. JL, AA, FT, GD, LN, RG, RB, JH, RF, ST, DT, JR, AM, CO, SET, LA, TS, DT, CE, TC, CT, RP, GJ, SS, JH, AMC created meta-data, prepared or linked –data sources to create the cohort. JL, AA, FT, GD, LN, RG, RB, JH, RF, ST, DT, JR, AM, CO, SET, LA, LC, MG, SB, BL, AJ, TS, JD, CD, DRhT, CW, CE, SC, TC, CT, RP, PD, GJ, SS, JH, AMC, KH, RAL contributed to the drafting of the manuscript and gave final approval of the version to be published. RAL is the principle investigator and guarantor of the study.

Acknowledgements

The authors would like to acknowledge that this work uses data provided by patients and collected by the NHS as part of their care and support and the Understanding Patient Data initiative. We would also like to acknowledge all data providers who make anonymised data available for research.

We wish to acknowledge the collaborative partnership that enabled acquisition and access to the de-identified data, which led to this output. The collaboration was led by the Swansea University Health Data Research UK team under the direction of the Welsh Government Technical Advisory Group (TAG). The team includes the following groups and organisations: the Secure Anonymised Information Linkage (SAIL) Databank, Administrative Data Research (ADR) Wales, NHS Wales Informatics Service (NWIS), Public Health Wales, NHS Shared Services and the Welsh Ambulance Service Trust (WAST). All research conducted has been completed under the permission and approval of the SAIL independent Information Governance Review Panel (IGRP) project number 0911.

Funding

The WMC, on which components of this study are based, is funded by Health Data Research UK (HDR-9006) and the Medical Research Council (MR/S027750/1). Funding for the COVID extension is through the Medical Research Council (MR/V028367/1). Health Data Research UK is funded by: UK Medical Research Council; Engineering and Physical Sciences Research Council; Economic and Social Research Council; National Institute for Health Research (England); Chief Scientist Office of the Scottish Government Health and Social Care

For peer review only

Directorates; Health and Social Care Research and Development Division (Welsh Government); Public Health Agency (Northern Ireland); British Heart Foundation; and Wellcome.

Disclaimer

The views and opinions expressed therein are those of the authors and do not necessarily reflect those of the funding agencies, NHS organisations or Welsh Government.

Competing interests

None declared.

Provenance and peer review

The WMC was peer–reviewed and specific objectives funded by the Medical Research Council (MR/S027750/1). This COVID19 extension has been peer-reviewed and an award will be made by a major UK research funder. Details will be included as soon as the funder makes an official announcement.

Figure captions

Figure 1: Data linkage of multiple demographic and healthcare data sources used in the creation of two population wide cohorts: C20 and C16

Figure 2: CONSORT diagram of the C20 cohort inclusion criteria

For peer review only

References

1. COVID-19 Dashboard by the Centre for Systems Science and Engineering (CSSE) at Johns Hopkins University (JHU). Available: https://coronavirus.jhu.edu/map.html [Accessed 1 June 2020].
2. Lyons R, Jones K, John G, Brooks C, Verplancke J, Ford D et al. The SAIL databank: linking multiple health and social care datasets. *BMC Medical Informatics and Decision Making* 2009;9(1).
3. Ford DV, Jones KH, Verplancke J-P, Lyons RA, John G, Brown G, et al. The SAIL Databank: building a national architecture for e-health research and evaluation. *BMC Health Services Research* 2009;9(1).
4. Lyons RA, Ford DV, Moore L, Rodgers SE. Use of data linkage to measure the population health effect of non-health-care interventions. The Lancet. 2014;383(9927):1517–9.
5. Jones KH, Ford DV, Jones C, Dsilva R, Thompson S, Brooks CJ, et al. A case study of the Secure Anonymous Information Linkage (SAIL) Gateway: A privacy-protecting remote access system for health-related research and evaluation. Journal of Biomedical Informatics. 2014;50:196–204.
6. Lyons RA, Turner S, Lyons J, Walters A, Snooks HA, Greenacre J, et al. All Wales Injury Surveillance System revised: development of a population-based system to evaluate single-level and multilevel interventions. Injury Prevention. 2015;22(Suppl 1):i50–i55.
7. Snooks HA, Anthony R, Chatters R, Dale J, Fothergill R, Gaze S, et al. Support and Assessment for Fall Emergency Referrals (SAFER) 2: a cluster randomised trial and systematic review of clinical effectiveness and cost-effectiveness of new protocols for emergency ambulance paramedics to assess older people following a fall with referral to community-based care when appropriate. Health Technology Assessment. 2017;21(13):1–218.
8. Hollinghurst J, Akbari A, Fry R, Watkins A, Berridge D, Clegg A, et al. Study protocol for investigating the impact of community home modification services on hospital utilisation for fall injuries: a controlled longitudinal study using data linkage. BMJ Open. 2018;8(10).
9. Mizen A, Song J, Fry R, Akbari A, Berridge D, Parker SC, et al. Longitudinal access and exposure to green-blue spaces and individual-level mental health and well-being: protocol for a longitudinal, population-wide record-linked natural experiment. BMJ Open. 2019;9(4).

For peer review only

10. Szakmany T, Walters AM, Pugh R, Battle C, Berridge DM, Lyons RA. Risk Factors for 1-Year Mortality and Hospital Utilization Patterns in Critical Care Survivors. Critical Care Medicine. 2019;47(1):15–22.

11. Rodgers SE, Bailey R, Johnson R, Berridge D, Poortinga W, Lannon S, et al. Emergency hospital admissions associated with a non-randomised housing intervention meeting national housing quality standards: a longitudinal data linkage study. Journal of Epidemiology and Community Health. 2018;72(10):896–903.

12. Paranjothy S, Evans A, Bandyopadhyay A, Fone D, Schofield B, John A, et al. Risk of emergency hospital admission in children associated with mental disorders and alcohol misuse in the household: an electronic birth cohort study. The Lancet Public Health. 2018;3(6).

13. Schnier C, Wilkinson T, Akbari A, Orton C, Sleegers K, Gallacher J, et al. Cohort profile: The Secure Anonymised Information Linkage databank Dementia e-cohort (SAIL-DeC). International Journal of Population Data Science. 2020;5(1).

14. Rodgers SE, Lyons RA, Dsilva R, Jones KH, Brooks CJ, Ford DV, et al. Residential Anonymous Linking Fields (RALFs): a novel information infrastructure to study the interaction between the environment and individuals' health. Journal of Public Health. 2009;31(4):582–8.

15. Welsh Index Multiple Deprivation Index. Available: https://gov.wales/welsh-index-multiple-deprivation-index-guidance [Accessed 9 April 2020].

16. 2011 rural/urban classifications. Available: https://www.ons.gov.uk/methodology/geography/geographicalproducts/ruralurbanclassifications/2011ruralurbanclassification [Accessed 9 April 2020].

17. Welsh Government. Pupil level annual school census (PLASC). Available: https://gov.wales/pupil-level-annual-school-census-plasc [Accessed 14 June 2020].

18. Welsh Government. School Workforce Annual Census. Welsh Government. Available: https://gov.wales/school-workforce-annual-census- [Accessed 16 June 2020].

19. Office of National Statistics. 2011 Census analysis: ethnicity and religion of the non-UK born population in England and Wales: 2011. Available: https://www.ons.gov.uk/peoplepopulationandcommunity/culturalidentity/ethnicity/articles/2011censusanalysisethnicityandreligionofthenonukbornpopulationinenglandandwales/2015-06-18 [Accessed 16 June 2020].

20. Thayer D, Rees A, Kennedy J, Collins H, Harris D, Halcox J, et al. Measuring follow-up time in routinely-collected health datasets: Challenges and solutions. Plos One. 2020;15(2).

For peer review only

21. Menni, C., Valdes, A.M., Freidin, M.B. *et al.* Real-time tracking of self-reported symptoms to predict potential COVID-19. *Nat Med* (2020). https://doi.org/10.1038/s41591-020-0916-2
22. Intensive Care National Audit and Research Centre (ICNARC). ICNARC report on COVID-19 in critical care in Wales. *Available:* https://www.icnarc.org/Our-Audit/Audits/Cmp/Reports *[Accessed* 02 July 2020*].*
23. Intensive Care National Audit and Research Centre (ICNARC). ICNARC report on COVID-19 in critical care 26 June 2020*. Available:* https://www.icnarc.org/Our-Audit/Audits/Cmp/Reports *[Accessed* 02 July 2020*].*
24. An integrated national scale SARS-CoV-2 genomic surveillance network. The Lancet Microbe. 2020;1(3).
25. NHS Wales Shared Services. Community pharmacy dispensing data. Available: https://nwssp.nhs.wales/ourservices/primary-care-services/general-information/data-and-publications/pharmacy-practice-dispensing-data/ [Accessed 24 June 2020].
26. Office for National Statistics. Ethnicity, disability, religion. Which groups are most at risk of death involving COVID19? Available: https://blog.ons.gov.uk/2020/06/19/ethnicity-disability-religion-which-groups-are-at-most-risk-of-death-involving-covid-19/ [Accessed 24 June 2020].
27. Harrison EM, Docherty AB, Barr B, Buchan I, Carson G, Drake TM, et al. Ethnicity and Outcomes from COVID-19: The ISARIC CCP-UK Prospective Observational Cohort Study of Hospitalised Patients. SSRN Electronic Journal. 2020;
28. Docherty AB, Harrison EM, Green CA, Hardwick HE, Pius R, Norman L, et al. Features of 20 133 UK patients in hospital with covid-19 using the ISARIC WHO Clinical Characterisation Protocol: prospective observational cohort study. Bmj. 2020;:m1985.
29. Kuan V, Denaxas S, Gonzalez-Izquierdo A, Direk K, Bhatti O, Husain S, et al. A chronological map of 308 physical and mental health conditions from 4 million individuals in the English National Health Service. The Lancet Digital Health. 2019;1(2).
30. Charlson ME, Pompei P, Ales KL, Mackenzie C. A new method of classifying prognostic comorbidity in longitudinal studies: Development and validation. Journal of Chronic Diseases. 1987;40(5):373–83.
31. Thompson NR, Fan Y, Dalton JE, Jehi L, Rosenbaum BP, Vadera S, et al. A New Elixhauser-based Comorbidity Summary Measure to Predict In-Hospital Mortality. Medical Care. 2015;53(4):374–9.

For peer review only

32. Hollinghurst J, Fry R, Akbari A, Clegg A, Lyons RA, Watkins A, et al. External validation of the electronic Frailty Index using the population of Wales within the Secure Anonymised Information Linkage Databank. Age and Ageing. 2019;48(6):922–6.
33. Gilbert T, Neuburger J, Kraindler J, Keeble E, Smith P, Ariti C, et al. Development and validation of a Hospital Frailty Risk Score focusing on older people in acute care settings using electronic hospital records: an observational study. The Lancet. 2018;391(10132):1775–82.
34. Clegg A, Bates C, Young J, Ryan R, Nichols L, Teale EA, et al. Development and validation of an electronic frailty index using routine primary care electronic health record data. Age and Ageing. 2017;
35. Simpson CR, Robertson C, Vasileiou E, Mcmenamin J, Gunson R, Ritchie LD, et al. Early Pandemic Evaluation and Enhanced Surveillance of COVID-19 (EAVE II): protocol for an observational study using linked Scottish national data. BMJ Open. 2020;10(6).
36. Victora CG, Huttly SR, Fuchs SC, Olinto MT. The role of conceptual frameworks in epidemiological analysis: a hierarchical approach. International Journal of Epidemiology. 1997;26(1):224–7.
37. R Studio Team (2020). RStudio: Integrated Development for R. RStudio, PBC, Boston, MA URL. Available: http://www.rstudio.com/.
38. R Core Team (2019). R: A language and environment for statistical computing. R foundation for Statistical Computing, Vienna, Austria. Available: http://www.R-project.org/.
39. Petersen I, Douglas I, Whitaker H. Self controlled case series methods: an alternative to standard epidemiological study designs. Bmj. 2016;:i4515.

For peer review only

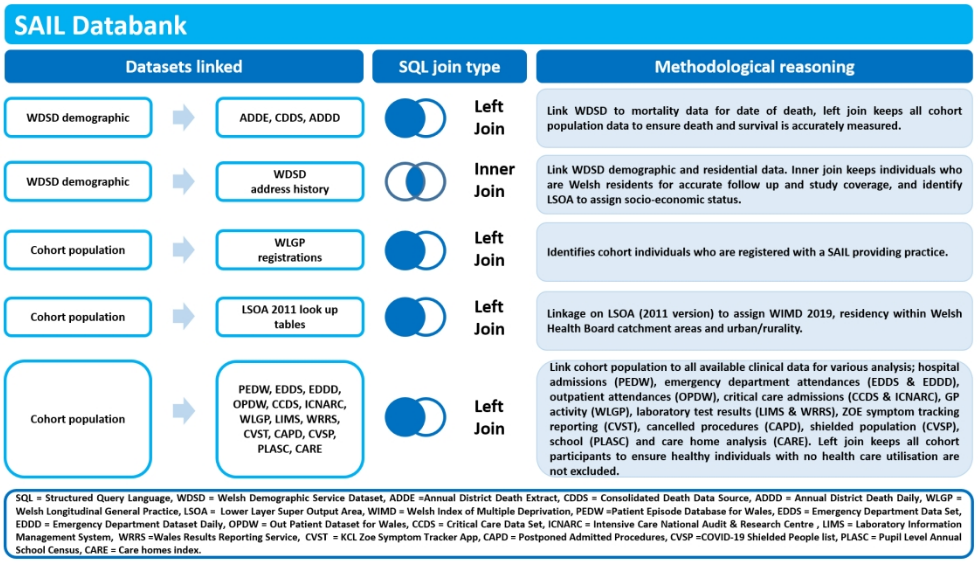

Figure 1: Data linkage of multiple demographic and healthcare data sources used in the creation of two population wide cohorts: C20 and C16.

342x194mm (150 x 150 DPI)

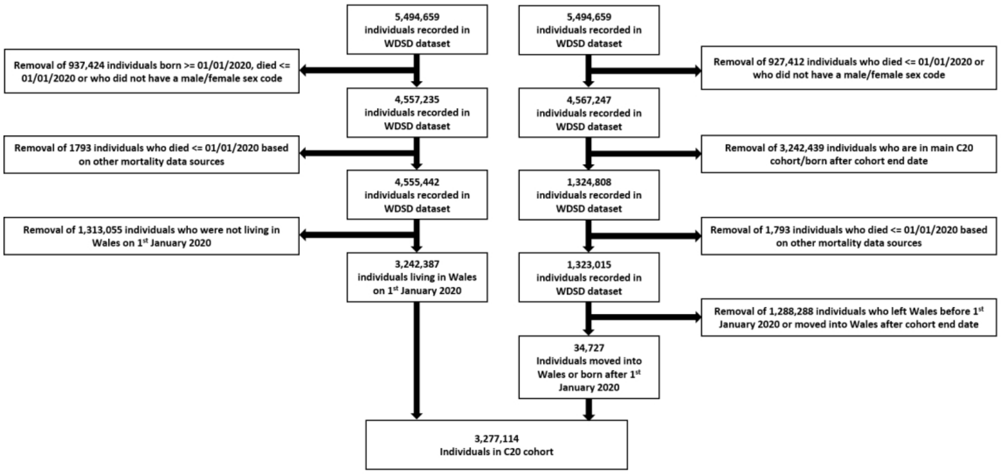

Figure 2: CONSORT diagram of the C20 cohort inclusion criteria

287x137mm (150 x 150 DPI)
